# Risk of Early Deterioration in Emergency Department Patients Presenting with Non-Massive Hemoptysis: A Prospective Cohort Study †

**DOI:** 10.3390/jcm15156004

**Published:** 2026-08-02

**Authors:** Mutlu Onur Güçsav, Onur Akçay, Hakan Alkan, Beril Aleyna Genç, Mukaddes Hande Özgen, Aysu Ayrancı, Ahmet Emin Erbaycu

**Affiliations:** 1 Department of Pulmonology, Cigli Training and Research Hospital, Izmir Bakırçay University, 35620 Izmir, Türkiye; hakanalkan96@gmail.com (H.A.); aysu.ayranci@bakircay.edu.tr (A.A.); ahmet.erbaycu@bakircay.edu.tr (A.E.E.); 2 Translational Pulmonary Research Center (EgeSAM), Ege University, 35100 Izmir, Türkiye; 3 Department of Thoracic Surgery, Cigli Training and Research Hospital, Izmir Bakırçay University, 35620 Izmir, Türkiye; onurakcay82@gmail.com; 4 Department of Anesthesiology and Intensive Care, Dokuz Eylül University, 35320 Izmir, Türkiye; beril.genc.1@gmail.com; 5 Department of Occupational and Environmental Medicine, Cigli Training and Research Hospital, Izmir Bakırçay University, 35620 Izmir, Türkiye; handebahadir86@gmail.com

**Keywords:** hemoptysis, risk factor, clinical prognosis, emergency department, risk stratification

## Abstract

**Background:** Non-massive hemoptysis is generally considered low-risk and manageable with conservative treatment. However, some patients progress to massive hemoptysis during follow-up. Identifying high-risk patients early in the emergency department matters for calibrating monitoring intensity, guiding timely intervention, and allocating acute care resources. This study aimed to identify clinical, laboratory, and radiological predictors of progression to massive hemoptysis within the first 72 h in emergency department patients presenting with non-massive hemoptysis who were managed conservatively. **Methods:** This prospective cohort study enrolled patients at a tertiary university hospital emergency department between November 2023 and June 2025. Adult patients presenting with non-massive hemoptysis were enrolled consecutively. The primary outcome was development of massive hemoptysis within 72 h of admission. Patients were divided into two groups: those who developed massive hemoptysis within 72 h and those who did not. Demographic, bleeding, laboratory, imaging, and bronchoscopy data were recorded for all patients. Multivariate logistic regression was used to identify independent predictors. **Results:** Of 199 patients, 10.6% developed massive hemoptysis within the first 72 h. On multivariate analysis, bright red hemoptysis (3.17-fold increase in risk), a cavity or mass on thoracic CT (7.13-fold increase in risk), and bleeding volume ≥20 mL in a single episode (3.3-fold increase in risk) were independent predictors of massive hemoptysis. **Conclusions:** A meaningful proportion of patients presenting with non-massive hemoptysis go on to develop massive hemoptysis in the early period. Simple clinical and radiological parameters available at admission can support early risk stratification and inform decisions regarding monitoring intensity and early inpatient management during the critical first 72 h after emergency department admission. These findings may assist early risk stratification but should complement, rather than replace, clinical judgement.

## 1. Introduction

Hemoptysis, the expectoration of blood from the lung parenchyma or tracheobronchial tree, is a common emergency department admission and a significant diagnostic challenge for clinicians. Severity is most commonly gauged by the volume of blood expectorated in the preceding 24 h, and bleeding is broadly divided into two categories: non-massive (mild to moderate) hemoptysis and life-threatening massive hemoptysis [1,2,3].

However, the definition of massive hemoptysis remains highly heterogeneous in the literature. Reported threshold volumes range from 100 to 1000 mL over 24 h [4,5,6,7,8,9,10]. This variability reflects differences in clinical context, study populations, and how severity itself has been conceptualized over time. Some expert recommendations shift the focus from absolute volume to clinical impact, defining massive hemoptysis by its life-threatening consequences. Under this framework, massive hemoptysis encompasses bleeding that becomes life-threatening due to airway obstruction, hypoxemia, hemodynamic instability, or the need for interventions such as blood transfusion, hospitalization, or mechanical ventilation [10,11,12]. Either way, the clinical impact of bleeding may matter as much as the volume itself when assessing patient risk.

Massive hemoptysis remains one of the most feared emergencies in pulmonary and emergency medicine, given its potential to cause life-threatening complications such as airway obstruction and respiratory failure. Although it accounts for only about 5% of all hemoptysis cases, reported mortality rates range from 6.5% to 38% [13,14,15]. Most cases originate from the bronchial circulation; bleeding under systemic arterial pressure can turn life-threatening within minutes [16]. In the emergency department, distinguishing patients likely to remain stable from those at risk of rapid deterioration is therefore a real challenge. Identifying high-risk patients early is essential for timely decisions about monitoring, admission, and treatment.

Unlike massive hemoptysis, non-massive hemoptysis is generally considered low-risk and is usually managed conservatively. However, its clinical course can be unpredictable, and it may be the first sign of a serious underlying disease [1,3,17,18]. Prior studies have shown that recurrence rates can reach 17–19% over long-term follow-up, and some patients go on to develop increasingly severe bleeding episodes [12,19]. These findings suggest that non-massive hemoptysis should not always be treated as a benign condition.

Although existing clinical scoring systems aim to predict early poor outcomes, no prospective data evaluate progression from non-massive to massive hemoptysis within the first 72 h of admission [1,18]. This gap is particularly relevant because the first 72 h represent the period during which most decisions regarding hospitalization, discharge, monitoring intensity, and escalation of care are made. Identifying patients at risk of early deterioration during this interval may therefore directly support emergency department management.

This study aimed to assess how often non-massive hemoptysis progresses to massive hemoptysis within the first 72 h in emergency department patients, and to identify the clinical, laboratory, and radiological risk factors associated with that progression.

## 2. Material and Methods

### 2.1. Study Design and Setting

This prospective cohort study was conducted in the emergency department of a university hospital in Turkey between 1 November 2023 and 1 June 2025, with follow-up carried out in collaboration with the pulmonology department. The study protocol was reviewed and approved by the institutional ethics committee (Approval No: 1235, 11 October 2023). The study was conducted in accordance with the ethical principles of the 1964 Declaration of Helsinki and its subsequent amendments. Written informed consent was obtained from all participants prior to enrollment.

All adult patients presenting to the emergency department with hemoptysis during the study period were consecutively evaluated (*n* = 278). Excluded were patients with a prior history of massive hemoptysis (*n* = 16), those with bleeding from the oral cavity, upper airway, or gastrointestinal tract that could mimic hemoptysis (*n* = 48), those with an international normalized ratio (INR) >2 (*n* = 10), and individuals with missing data (*n* = 5). After applying these exclusion criteria, 199 patients who completed all clinical assessments were included in the final analysis. Patients were followed prospectively for 30 days. The primary outcome was development of massive hemoptysis within the first 72 h after emergency department admission. The 72 h endpoint was prospectively defined before patient enrollment because this interval represents the clinically most relevant period for early deterioration after emergency department admission. During this interval, decisions regarding hospitalization, discharge, monitoring intensity, and therapeutic escalation are typically made in routine clinical practice.

The dataset included demographic characteristics, medical history, laboratory findings, flexible fiberoptic bronchoscopy (FOB) findings, multidetector thoracic computed tomography (CT) and CT pulmonary angiography (CTPA) findings, bleeding volume and characteristics, hemoptysis recurrence, hospitalization, interventional procedures, and early and late prognostic outcomes.

### 2.2. Definitions and Assessment of Bleeding Volume

Massive and non-massive hemoptysis were classified using volume-based thresholds widely used in the literature [4,5,6,7,8,9,10]. Bleeding exceeding 400 mL in the 24 h before admission was defined as massive hemoptysis; bleeding ≤400 mL over the same period was classified as non-massive. Although physiology-based definitions of life-threatening hemoptysis have gained increasing acceptance because they better reflect the clinical consequences of bleeding, a volume-based threshold of >400 mL/24 h was selected a priori for this study to provide an objective, reproducible, and standardized primary outcome definition. Furthermore, this threshold remains one of the most frequently used volume-based definitions in prospective clinical studies, facilitating comparison with previously published literature.

To improve standardization of patient-reported bleeding volume estimates, household volume references were used. Patients were shown common household measures, including a teaspoon (2.5 mL), dessertspoon (5 mL), tablespoon (10 mL), tea glass (100 mL), and water glass (200 mL), and asked to identify which container and what proportion of it best matched their total expectorated blood volume. Although objective measurement of expectorated blood volume is not feasible in routine emergency department practice, the use of standardized household volume references was intended to improve consistency across patient estimates.

### 2.3. Imaging Techniques

All posteroanterior chest radiographs were acquired by radiologic technologists with a minimum of three years of experience using a Samsung^®^ XGEO GC80 system (Samsung Electronics Corp. Ltd., Suwon, Republic of Korea), in accordance with the European Guidelines on Quality Criteria for Diagnostic Radiographic Image [20]. Thoracic CT examinations were performed by technologists with at least three years of experience using a GE Optima^®^ CT660^®^ 128-detector spiral CT scanner (GE HealthCare Corp., Hino/Tokyo, Japan) [21]. For patients undergoing CTPA, 50 mL of an iohexol-based intravenous contrast agent was administered as a bolus through an antecubital vein, followed by a saline flush at a rate of 4.5 mL/s.

### 2.4. Bronchoscopy Standardization

All fiberoptic bronchoscopy procedures were performed by two experienced bronchoscopists using a Fujifilm^®^ EB-580T flexible fiberoptic bronchoscope (Fujifilm Corp., Tokyo, Japan). The procedure was performed for diagnostic purposes, to identify the bleeding source and evaluate endobronchial pathologies, with no intent for emergency therapeutic intervention. In most cases, bronchoscopy was performed electively based on clinical assessment. Active bleeding, bloody secretions, clots, and other endobronchial findings were systematically recorded during bronchoscopy.

### 2.5. Follow-Up and Primary Outcome

**Figure 1:**
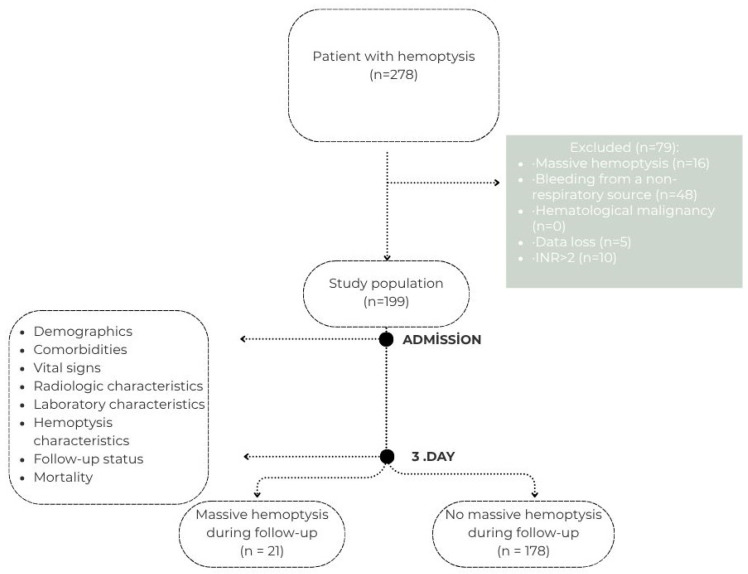
Flow chart.

Although follow-up continued for the full 30 days, development of massive hemoptysis within the first 72 h of admission was prospectively pre-specified as the primary outcome because this interval represents the clinically most relevant period for early deterioration following emergency department admission. Patients were divided into two groups based on whether massive hemoptysis developed within this period. The two groups were compared according to clinical characteristics, laboratory parameters, and imaging findings (Figure 1).

### 2.6. Statistical Analysis

Statistical analyses were performed using SPSS version 25 (IBM Corp., Armonk, NY, USA). Categorical variables are summarized as counts and percentages; continuous variables are reported as mean ± standard deviation or median (25th–75th percentile) depending on their distribution. The distribution of continuous variables was assessed using the Kolmogorov–Smirnov test. For between-group comparisons of categorical variables, the chi-square test was used; Fisher’s exact test was applied when chi-square assumptions were not met. For multi-cell contingency tables where the Pearson chi-square test was inappropriate, Fisher’s exact test with Monte Carlo simulation was used and two-sided Monte Carlo significance values were reported. Non-normally distributed continuous variables were compared using the Mann–Whitney U test. A *p*-value < 0.05 was considered statistically significant.

Multivariate logistic regression analysis was performed to identify independent predictors of massive hemoptysis within the first 72 h. Variables significant at *p* < 0.10 in univariate analyses were included as candidates for the multivariate model. To reduce the risk of multicollinearity, only one variable from each pair representing the same clinical construct or showing high intercorrelation was retained in the model (e.g., INR/PT, total 24 h bleeding volume/single-episode bleeding volume, SpO_2_/oxygen requirement). All candidate variables were entered into the full model initially, and the final model was built using purposeful backward elimination.

Because only 21 primary outcome events occurred, particular attention was paid to minimize model overfitting. Candidate variables were first screened using univariable analyses, and clinically correlated variables were not entered simultaneously into the multivariable logistic regression model. The final multivariable model retained only three independent predictors, corresponding to an events-per-variable ratio of approximately seven. Although this ratio is considered acceptable for exploratory multivariable regression analyses, the findings should be interpreted with caution and require validation in independent cohorts.

## 3. Results

Of the 199 patients included, 68.3% (*n* = 136) were male, with a mean age of 59.4 ± 16.3 years. Active smoking was present in 42.2% (*n* = 84), while 19.1% (*n* = 38) had never smoked. At least one chronic comorbidity was present in 66.3% (*n* = 132); the most common were hypertension (43.7%), coronary artery disease (28.1%), and diabetes mellitus (24.1%). A history of tuberculosis was documented in 7.0% (*n* = 14). Anticoagulant therapy was used by 11.1% (*n* = 22), while 25.1% (*n* = 50) were on antiplatelet therapy.

62.8% of patients reported a first episode of hemoptysis. Recurrent bleeding during emergency department observation occurred in 33.7% (*n* = 67). 10.6% of patients developed massive hemoptysis within the first 72 h. On assessment of hemoptysis appearance, 45.7% (*n* = 91) described blood-streaked sputum, 43.7% (*n* = 87) reported bright red bleeding, and 10.6% (*n* = 21) reported dark red or black bleeding. The median time from bleeding onset to admission was 24 h (IQR: 8–48). The median blood volume expectorated in the 24 h before admission was 40 mL (IQR: 15–100), with patients reporting a median of five bleeding episodes (IQR: 3–5) over the same period. Median bleeding volume per episode was 10 mL (IQR: 5–20).

Pathological findings were present on posteroanterior chest radiographs in 58.8% (*n* = 117) of patients, and in 82.6% (*n* = 161) of the 195 who underwent thoracic CT angiography. The most common CT findings were ground-glass opacities (48.2%) and consolidation (22.1%). FOB was performed in 55.8% (*n* = 111) of patients; additional abnormal endobronchial findings beyond active bleeding were identified in 81.1% (*n* = 90) of these. The three most common bronchoscopic findings were residual blood (62.2%), petechiae (33.3%), and endobronchial lesions (16.2%).

The median hospital stay was 5 days (IQR: 2–8). Thirty-day mortality was 4.0% (*n* = 8). Of the eight deaths, four occurred in the massive hemoptysis group and four in those who did not develop it. Of these eight deaths, four were attributed to underlying malignancy, three to hospital-acquired infections, and one to acute renal failure. Recurrent bleeding was observed in 44.7% (*n* = 89) of patients within the first 72 h. Of these, 23.6% (*n* = 21) subsequently developed massive hemoptysis.

Among patients who developed massive hemoptysis within the first 72 h after admission, the median age was 59 years (IQR: 55–72), compared with 62 years (IQR: 47–70) in those who did not; however, this difference was not statistically significant (*p* = 0.952). Massive hemoptysis incidence was similar in women and men (9.5% vs. 11.0%, *p* = 0.748). On admission, patients who subsequently developed massive hemoptysis had significantly lower oxygen saturation (SpO_2_) and significantly higher heart rate compared with those who did not develop massive hemoptysis (*p* = 0.012 and *p* = 0.041, respectively). In addition, INR, prothrombin time (PT), and urea levels were significantly higher in patients who developed massive hemoptysis (*p* = 0.012, *p* = 0.023, and *p* = 0.047, respectively) (Table 1).

At admission, patients who subsequently developed massive hemoptysis within the first 72 h reported a higher median total bleeding volume during the preceding 24 h compared with those who did not develop massive hemoptysis (100 mL [IQR: 40–200] vs. 30 mL [IQR: 15–100], *p* = 0.003). The median bleeding volume per single episode at admission was also higher in patients who later progressed to massive hemoptysis (20 mL [IQR: 5–50] vs. 10 mL [IQR: 5–20], *p* = 0.008). These patients also had a longer median hospital stay (7 vs. 5 days, *p* = 0.002). Thirty-day mortality was significantly higher in patients who developed massive hemoptysis within the first 72 h (19.0% [*n* = 4] vs. 2.3% [*n* = 4], *p* < 0.001). Furthermore, intensive care unit admission was observed in 52.4% of patients with massive hemoptysis, compared with only 6.7% in those without massive hemoptysis (*p* < 0.001). Detailed information regarding bleeding characteristics and prognostic outcomes is presented in Table 2.

The two groups did not differ significantly in smoking status and comorbidity burden (*p* = 0.771 and *p* = 0.346, respectively). Pathological findings on posteroanterior chest radiograph were not significantly associated with massive hemoptysis (*p* = 0.438). In contrast, any pathological finding on thoracic CT, and cavitary and mass lesions in particular, were significantly associated with an increased risk of massive hemoptysis within the first 72 h (*p* = 0.028). In addition, the need for oxygen support at admission, bright red bleeding, and a history of previous hemoptysis were also significantly associated with massive hemoptysis during the first 72 h (*p* = 0.002, *p* = 0.008, and *p* = 0.045, respectively). The associations between clinical characteristics and the risk of massive hemoptysis within the first 72 h are detailed in Table 3.

On multivariate logistic regression, bright red hemoptysis, bleeding volume per episode, and a cavitary or mass lesion on thoracic CT were independent predictors of massive hemoptysis within the first 72 h after admission. Each 1 mL increase in bleeding volume per episode was associated with a 1.7% increase in the odds of developing massive hemoptysis. Total bleeding volume over 24 h, oxygen requirement at admission, history of hemoptysis, urea level, heart rate, and oxygen saturation (SpO_2_) were all entered into the multivariate model but none emerged as independent predictors. These findings are summarized in Table 4.

**Figure 2:**
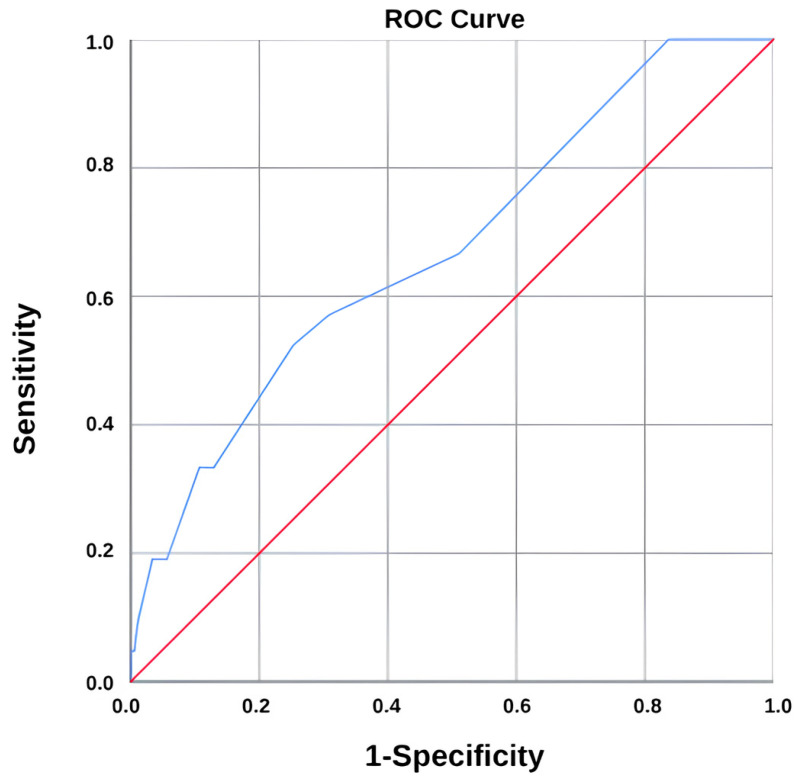
Receiver operating characteristic (ROC) curve illustrating the predictive performance of single-episode bleeding volume for massive hemoptysis.

ROC analysis identified an optimal threshold of ≥17.5 mL for bleeding volume per episode in predicting massive hemoptysis within the first 72 h (Youden index: 0.27) (Figure 2). At this threshold, sensitivity was 52.4% and specificity was 74.7%. Discriminative performance was moderate, with an area under the curve (AUC) of 0.672 (95% CI: 0.549–0.796). Since bleeding volume is generally estimated rather than precisely measured in routine clinical practice, a rounded threshold of 20 mL was selected as a more practical bedside reference rather than as a definitive clinical cutoff. Additional analyses showed that patients with a bleeding volume per episode of ≥20 mL had approximately 3.3 times higher odds of developing massive hemoptysis than those with <20 mL (OR = 3.25; 95% CI: 1.29–8.16).

## 4. Discussion

Hemoptysis is associated with a risk of recurrence and clinical deterioration even when the initial bleeding volume is limited. Previous studies have reported recurrence rates of up to 19% during long-term follow-up among patients presenting with mild hemoptysis, and approximately 9% of patients who re-present may subsequently develop massive hemoptysis [10,17,19]. These findings suggest that some patients initially classified as non-massive may progress to more severe bleeding over time. While prior studies have focused mainly on long-term outcomes, our findings show that progression to massive hemoptysis can occur as early as the first 72 h after admission. These findings indicate that initial bleeding volume alone may not adequately reflect the risk of early clinical deterioration, and highlight the importance of early risk stratification and close monitoring in the period immediately after admission. We specifically focused on the first 72 h because this period corresponds to the initial phase of emergency department management, during which clinicians determine the appropriate level of monitoring, need for hospitalization, and timing of further diagnostic or therapeutic interventions. Unlike studies evaluating long-term recurrence or mortality, our objective was to identify patients at risk of immediate clinical deterioration, thereby providing information directly applicable to early emergency department decision-making.

Studies evaluating long-term mortality in hemoptysis report an overall rate of approximately 13.7%, with lung malignancy as the strongest predictor [10]. The 30-day mortality rate in our study was comparable to the in-hospital rates reported by Hirshberg et al. and Fartoukh et al., despite differences in follow-up duration [8,22]. Mortality was significantly higher among patients who developed massive hemoptysis within the first 72 h than among those who did not. Although bleeding was not the direct cause of death in every case, early progression to massive hemoptysis appears to identify a subgroup with markedly worse short-term outcomes. Even in patients presenting with non-massive hemoptysis, the first 72 h therefore call for close monitoring and careful consideration of discharge decisions.

Variability in the emergency department management of hemoptysis, the lack of consensus regarding the definition of massive hemoptysis, and the inherent unreliability of bleeding volume estimation have led clinicians to favor risk stratification approaches based on “impact severity,” such as airway obstruction, hemodynamic instability, and the need for intensive care, rather than absolute volume alone [1,18]. Risk stratification tools such as the Florence Hemoptysis Score (FLHASc) have been developed to predict early poor outcomes. The FLHASc incorporates easily assessed variables including systolic blood pressure <100 mmHg, a history of malignancy, and more than two hemoptysis episodes in the preceding 24 h, and suggests that patients with a score of 0 and a normal chest radiograph can be safely discharged from the emergency department [1].

In the FLHASc study, 5.8% of patients discharged with mild hemoptysis returned to the emergency department within three months due to recurrent hemoptysis, and 1.5% re-presented within the first 72 h after discharge [1]. However, in that study and in others, the definition and timing of massive hemoptysis have generally relied on 24 h or longer windows, and none specifically reported the rate of progression from non-massive to massive hemoptysis [1,14,15,18]. In this respect, the present study distinguishes itself from prior work by providing original data on early progression from non-massive to massive hemoptysis.

In our study, bright red hemoptysis, higher bleeding volume per episode, and cavitary or mass lesions on thoracic CT were each independently associated with massive hemoptysis within the first 72 h. Unlike the FLHASc score, which primarily relies on readily available systemic clinical variables to predict early poor outcomes, our findings additionally incorporate bleeding characteristics and thoracic CT morphology. Rather than replacing existing risk stratification tools, these variables may provide complementary prognostic information, particularly in patients who undergo thoracic CT during emergency department evaluation. In routine emergency department practice, these variables may help determine the appropriate level of monitoring and patient disposition during the first 72 h after presentation, including whether patients can be safely discharged, hospitalized, or observed more closely. However, prospective external validation studies are required before these variables can be incorporated into routine clinical risk prediction algorithms.

Previous studies have proposed different threshold values for 24 h total bleeding volume as a prognostic marker in hemoptysis [1,23]. In the FLHASc study, prognosis was not associated with the total bleeding volume over 24 h but rather with the frequency of bleeding episodes during this period (>2 episodes/24 h) [1,11]. In our study, although a significant difference was observed between groups in terms of total bleeding volume during the 24 h preceding emergency department admission, this variable did not emerge as an independent predictor in multivariable analysis. Likewise, the number of bleeding episodes within 24 h was not associated with the development of massive hemoptysis. In contrast, a single-episode bleeding volume of ≥20 mL was associated with an approximately threefold increase in the odds of developing massive hemoptysis within the first 72 h. These findings suggest that the volume of bleeding during an individual episode may contribute additional prognostic information beyond total 24 h bleeding volume and episode frequency. Because bleeding volume was based on patient estimates, the proposed threshold should be interpreted as an approximate clinical indicator rather than an exact quantitative measurement. Despite this limitation, estimation of bleeding volume based on patient history and standardized household volume references reflects routine emergency department practice, where objective quantification of expectorated blood is rarely feasible. However, the discriminatory performance of the proposed ≥20 mL threshold was only moderate, indicating that it should not be interpreted as a standalone clinical decision threshold. Therefore, this practical bedside threshold may assist clinical assessment when considered together with radiological findings, bleeding characteristics, and overall clinical judgement.

Macroscopic characteristics of hemoptysis, including color, appearance, and odor, may provide insights not only into etiology but also into prognosis [24]. However, no study to date has directly assessed the relationship between the macroscopic characteristics of expectorated blood and clinical outcomes. In a study with a mean follow-up of approximately 2.5 years, the presence of active bleeding and residual blood on flexible fiberoptic bronchoscopy was shown to increase the risk of long-term recurrence by 3.3-fold and 2.7-fold, respectively [19]. In our study, bronchoscopic findings were not associated with early progression to massive hemoptysis; however, bright red hemoptysis emerged as an independent predictor within the first 72 h. Bright red blood at admission likely reflects more active or ongoing bleeding, which may explain this finding. Simple visual features of hemoptysis may therefore carry clinically meaningful prognostic information and should be factored into the initial assessment of patients presenting to the emergency department.

Pulmonary tuberculosis and bronchogenic carcinoma are well-known causes of massive hemoptysis. One reason is that both can produce cavitary or mass lesions in the lung parenchyma, which raises the risk of serious bleeding [14,25]. In keeping with this, our study found that cavitary or mass lesions on thoracic CT were independently associated with massive hemoptysis within the first 72 h. These findings suggest that these CT features may carry value not only for identifying the underlying etiology but also for early prognostic assessment. Although thoracic CT was performed in nearly all patients, imaging was obtained according to routine clinical practice rather than a predefined research protocol. Therefore, while the likelihood of substantial selection bias is low, it cannot be completely excluded.

Although SpO_2_ has not been consistently shown as an independent predictor of outcomes in hemoptysis, respiratory failure and the need for mechanical ventilation have been linked to poor prognosis in several studies [1,19,22]. In our cohort, SpO_2_ at admission was significantly lower in patients who subsequently developed massive hemoptysis. However, SpO_2_ did not emerge as an independent predictor in multivariate analysis. This aligns with prior work and suggests that oxygen desaturation reflects the physiological effects of bleeding rather than independently driving clinical deterioration. That said, SpO_2_ is quick to measure at the bedside and can add useful clinical context during the initial assessment of patients presenting with hemoptysis.

Uremia impairs hemostasis by disrupting platelet function and endothelial integrity, and bleeding complications are more common in patients with elevated urea levels [26]. In our study, urea levels were significantly higher in patients who developed massive hemoptysis within the first 72 h, but this association did not survive multivariate analysis after adjusting for other clinical variables. Elevated urea may reflect a higher-risk clinical profile overall; our findings, however, do not support it as an independent predictor of early progression to massive hemoptysis.

## 5. Conclusions

Patients presenting with non-massive hemoptysis remain at risk of early progression to massive hemoptysis despite an initially stable admission. In this prospective cohort, bright red hemoptysis, a single-episode bleeding volume of ≥20 mL, and cavitary or mass lesions on thoracic CT were independently associated with early deterioration within the first 72 h. These readily available clinical and radiological findings may assist clinicians in identifying patients who require closer monitoring and timely escalation of care during the early phase of emergency department management. However, these findings should be interpreted as complementary to clinical judgement and require prospective multicenter validation before routine clinical implementation.

## 6. Limitations

This study has several limitations. First, this was a single-center study conducted at a tertiary referral university hospital. Consequently, the study population may have included a greater proportion of patients with complex underlying diseases and more severe clinical presentations than would typically be encountered in community hospitals or secondary-care emergency departments. In addition, diagnostic resources and specialist availability at tertiary centers may differ from those in other healthcare settings. Therefore, the generalizability of our findings to different emergency care environments should be interpreted with caution. Second, the relatively small number of patients who developed massive hemoptysis within the first 72 h may have reduced statistical power and introduced greater uncertainty into the multivariate estimates. Nevertheless, the relatively limited number of outcome events may still have affected coefficient stability, and the model should therefore be externally validated in larger cohorts.

Because bleeding volume was estimated through patient self-report despite the use of standardized household volume references, recall bias and subjective estimation were unavoidable. Consequently, some degree of non-differential measurement error may have occurred, potentially attenuating the observed associations rather than artificially strengthening them. Nevertheless, this approach reflects routine emergency department practice, where bleeding volume is rarely measured objectively and clinical decisions frequently rely on patient-reported estimates.

Bronchoscopy was performed according to clinical indication rather than a predefined protocol, and therefore patients perceived to be at higher clinical risk were more likely to undergo the procedure. This may have introduced selection bias and limited the interpretation of bronchoscopic findings. Likewise, although thoracic CT was obtained in nearly all patients, imaging decisions also reflected routine clinical practice rather than a standardized research protocol, and a small degree of selection bias cannot be completely excluded. The >400 mL/24 h threshold used to define massive hemoptysis was chosen from among the heterogeneous definitions in the literature, which may limit direct comparability with studies using different criteria.

Finally, given the observational design, the associations identified should be interpreted as associative rather than causal. Because the primary objective of this study was the exploratory identification of independent predictors rather than the development of a definitive clinical prediction model, formal internal validation procedures such as bootstrap resampling were not performed. Therefore, both internal validation and external validation in larger multicenter cohorts, including primary-, secondary-, and tertiary-care emergency departments, will be necessary before these findings can be incorporated into routine clinical risk stratification. Accordingly, the results should be interpreted as exploratory hypothesis-generating findings rather than as a definitive clinical prediction model.

## Figures and Tables

**Table 1:** Comparison of laboratory and vital parameters between the groups.

Variables (Median [IQR])	No Massive Hemoptysis (*n* = 178)	Massive Hemoptysis (*n* = 21)	*p* Value
Systolic blood pressure (mmHg)	130 (120–152)	137 (100–157)	0.904
Diastolic blood pressure (mmHg)	75 (70–80)	80 (66–90)	0.576
Heart rate (beats/min)	85 (80–95)	94 (82–110)	0.041
SpO_2_ (%)	97 (95–98)	95 (88–97)	0.012
White blood cell count (cells/µL)	8550 (7230–10,650)	8240 (7470–11,400)	0.997
Neutrophil count (cells/µL)	5540 (4250–7900)	5660 (4770–6270)	0.966
Lymphocyte count (cells/µL)	1860 (1360–2440)	1600 (1020–2320)	0.388
Hemoglobin (g/dL)	13.1 (11.3–14.3)	12.6 (11.2–14.5)	0.778
Hematocrit (%)	39.2 (35.4–42.8)	38.5 (34.7–43.6)	0.949
Platelet count (×10^3^/µL)	253 (200–299)	234 (209–401)	0.994
Urea (mg/dL)	32 (25–41.5)	37.3 (29.4–56.0)	0.047
AST (U/L)	18 (14–23)	15 (13–23)	0.445
ALT (U/L)	14 (10–22)	15 (12–20)	0.997
CRP (mg/L)	8 (2.5–39.3)	8 (3.8–47.1)	0.533
INR	1.0 (0.9–1.1)	1.1 (1.0–1.2)	0.012
PT (s)	12.2 (11.5–13.2)	13.1 (12.3–14.0)	0.023
aPTT (s)	25.2 (23.4–27.1)	26.4 (24.4–28.3)	0.242

SpO_2_: Oxygen saturation, AST: Aspartate aminotransferase, ALT: Alanine aminotransferase, CRP: C-reactive protein, INR: International normalized ratio, PT: Prothrombin time, aPTT: Activated partial thromboplastin time, IQR: Interquartile range.

**Table 2:** Comparison of bleeding characteristics and length of hospital stay between the groups.

Variables (Median [IQR])	No Massive Hemoptysis (*n* = 178)	Massive Hemoptysis (*n* = 21)	*p* Value
Total bleeding volume within 24 h (mL)	30 (15–100)	100 (40–200)	0.003
Single-episode bleeding volume (mL)	10 (5–20)	20 (5–50)	0.008
Time since bleeding onset (hours)	22 (8–48)	24 (12–72)	0.151
Number of bleeding episodes,	5 (3–5)	4 (3–5)	0.683
Length of hospital stay (days)	5 (0–7)	7 (6–9)	0.002
30-day mortality, *n* (%)	4 (2.3)	4 (19.0)	<0.001

IQR: Interquartile range.

**Table 3:** Association between clinical characteristics and the risk of developing massive hemoptysis within the first 72 h.

Clinical Characteristics	Category	Massive Hemoptysis (*n* = 21), % (*n*)	*p* Value
O_2_ requirement at admission	No	7.8 (14)	0.002 †
	Yes	35.0 (7)	
Type of bleeding	Blood-streaked sputum	4.4 (4)	0.008 ‡
	Bright red blood	18.4 (16)	
	Dark red/black blood	4.8 (1)	
Presence of bleeding in the emergency department	No	7.6 (10)	0.055
	Yes	16.4 (11)	
History of hemoptysis	No	7.2 (9)	0.045
	Yes	16.2 (12)	
Pathological findings on chest X-ray	No	8.5 (7)	0.438
	Yes	12.0 (14)	
Pathological findings on thoracic CT	No	0	0.028 †
	Yes	13.0 (21)	
Cavitary lesion on thoracic CT	No	8.4 (15)	0.004 †
	Yes	35.3 (6)	
Mass lesion on thoracic CT	No	8.3 (13)	0.037 †
	Yes	21.1 (8)	

CT: computer tomography, O_2:_ Oxygen. † Fisher’s exact test was used. ‡ Due to low expected cell frequencies, the assumptions of the Pearson chi-square test were not met. Therefore, Fisher’s exact test with Monte Carlo simulation was applied, and the Monte Carlo two-sided significance value was reported.

**Table 4:** Multivariable logistic regression analysis for the development of massive hemoptysis.

Variable	OR	95% CI	*p* Value
Bright red hemoptysis	3.174	1.022–9.851	0.046
Single-episode bleeding volume (per mL)	1.017	1.004–1.031	0.012
Presence of cavitary or mass lesion on thoracic CT	7.135	2.060–24.715	0.002

CT: Computer tomography, OR: Odds ratio CI: Confidence interval. Hosmer–Lemeshow *p* = 0.240. Nagelkerke R^2^: 0.256. Reference category: dark red/black hemoptysis and blood-streaked sputum.

## Data Availability

The datasets generated and/or analyzed during the current study are available from the corresponding author on reasonable request.
